# Native tricuspid valve infective endocarditis with *Staphylococcus lugdunesis*: An unusual complication post spinal epidural injection – Case report and literature review

**DOI:** 10.1016/j.idcr.2021.e01097

**Published:** 2021-03-31

**Authors:** Osama Nweiran Al-Khalaila, Laith Fawzat Tbishat, Mohamed Salah Abdelghani, Ahmad A.A. Al Bishawi, Adila Shaukat Kashaf, Dina Alwaheidi, Abdulwahid Al Mulla

**Affiliations:** a Department of Cardiology, Heart Hospital, Hamad Medical Corporation, Qatar; b Department of Cardiothoracic Surgery, Heart Hospital, Hamad Medical Corporation, Qatar; c Department of Infectious Diseases, Hamad Medical Corporation, Qatar

**Keywords:** Infective endocarditis, Tricuspid valve, *Staphylococcus lugdunesis*, Coagulase negative staphylococcus, Epidural injection

## Abstract

Infective Endocarditis (IE) is a very rare complication following spinal epidural injection and requires high index of suspicion for early diagnosis and effective management.

*Staphylococcus Lugdunesis* is a coagulase negative staphylococcus (CoNS) that, unlike other CoNS, may result in aggressive form of native valve infective endocarditis (IE) mimicking IE caused by S aureus. Surgical intervention is usually needed to control infection in most cases of *S. Lugdunesis* IE.

Herein, we report a case of young lady with congenital Gerbode defect who developed tricuspid native valve IE with *S. Lugdunesis* secondary to spondylodiscitis post lumbar epidural injection that was performed for disk prolapse. She required urgent surgical intervention and had an excellent outcome.

## Introduction

Spinal epidural injection is a common and relatively safe procedure, however serious complications like abscess formation and IE may happen. S. lugdunensis is a newly recognized CoNS and considered as a normal skin flora, however it can result in a severe form of native valve endocarditis and usually requires surgical intervention. Early diagnosis and management are essential to avoid unfavorable outcome.

## Case presentation

A 38-year-old lady who has no past medical history of chronic illness apart from a congenital heart defect that was discovered incidentally during her childhood, she was told it is a small defect and does not require any further treatment or follow up (no physical documents or medical reports were available).

**Fig. 1:**
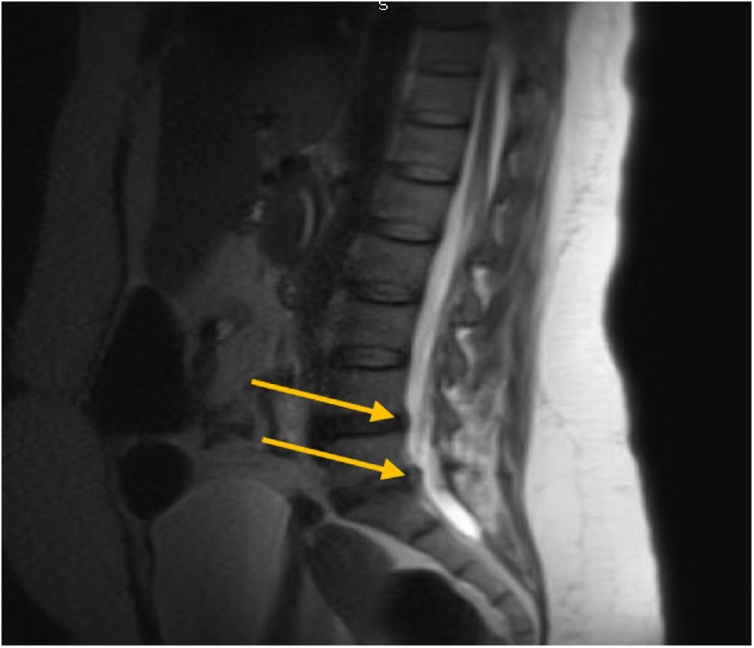
Initial Lumbosacral MRI showing mild impingement on the left L5 and S1 nerve roots.

The patient presented to the hospital with 3-month history of severe low back pain. MRI revealed L4/5 and L5/S1 left lateral disc protrusions with mild impingement on the left L5 and S1 nerve roots (Fig. 1). Patient underwent CT guided lumbar epidural injection with 80 mg methylprednisolone and 2 mL of 0.5 % marcaine. No immediate complications were encountered during or after the procedure.

Three weeks post procedure, patient presented to ED with 1-day history of chest pain and shortness of breath. She described the pain as progressive non-radiating left sided chest pain, dull in nature, constant and 5/10 in severity. There were no upper respiratory tract symptoms. Patient reported that she still has residual low back pain but less severe than it was before the procedure. On physical exam, patient was found to have fever of 38.3 ^c^ but otherwise she was hemodynamically stable with respiratory rate of 21 breath per minute and O_2_ saturation of 97 % on ambient air. The residual of her physical exam including cardiac exam was unremarkable. EKG showed normal sinus rhythm with no ST segment or T wave abnormalities. Lab tests were remarkable for high d-dimer (5.19 mg/L), other routine lab tests including WBC and Trop T were normal. Pulmonary embolism was suspected, so CT pulmonary angiogram (CTPA) was done and showed no evidence of pulmonary embolism, however, it showed small patchy consolidations involving multiple segments of the lungs on both sides. The patient was admitted with an impression of pneumonia and to rule out COVID-19 infection given her CT Findings, she received antibiotic treatment with cefuroxime and clarithromycin, COVID-19 PCR was negative, no blood cultures were sent, she was discharged home within few days after her symptoms improved.

Two weeks later, patient presented again to ED with 3-day history of fever, dry cough, fatigue and generalized body ache. On physical exam; blood pressure was 91/58 mmHg, pulse rate was 97 beat per minute, temperature was 38.1^c^, respiratory rate was 20 breath per minutes, O_2_ saturation 96 % on ambient air. Chest was clear on examination and cardiac exam did not reveal any murmur. Lab tests showed WBC 10.3 × 10^3^/uL, neutrophils: 76 %, C-reactive protein of 150 mg/L (normal < 5), her kidney function, liver function and serum electrolytes were normal. Subsequent lab tests for Brucella, COVID-19 PCR, HIV, Hepatitis B and C came back as negative. Chest X-ray showed small patchy opacity in the left upper zone. The patient was admitted again with an impression of community acquired pneumonia and she was started empirically on ceftriaxone and azithromycin. blood cultures were sent. Initial gram stain for blood cultures showed Gram positive cocci in cluster, subsequently vancomycin was added to her regimen. Later on, species identification reported coagulase negative staphylococcus. Final blood culture report showed *Staphylococcus Lugdunesis* growth, which was sensitive to Teicoplanin, Vancomycin, linezolid, Rifampicin, Trimethoprim/sulfamethoxazole and Clindamycin and resistant to cefazolin, cloxacillin and gentamycin.

**Fig. 2:**
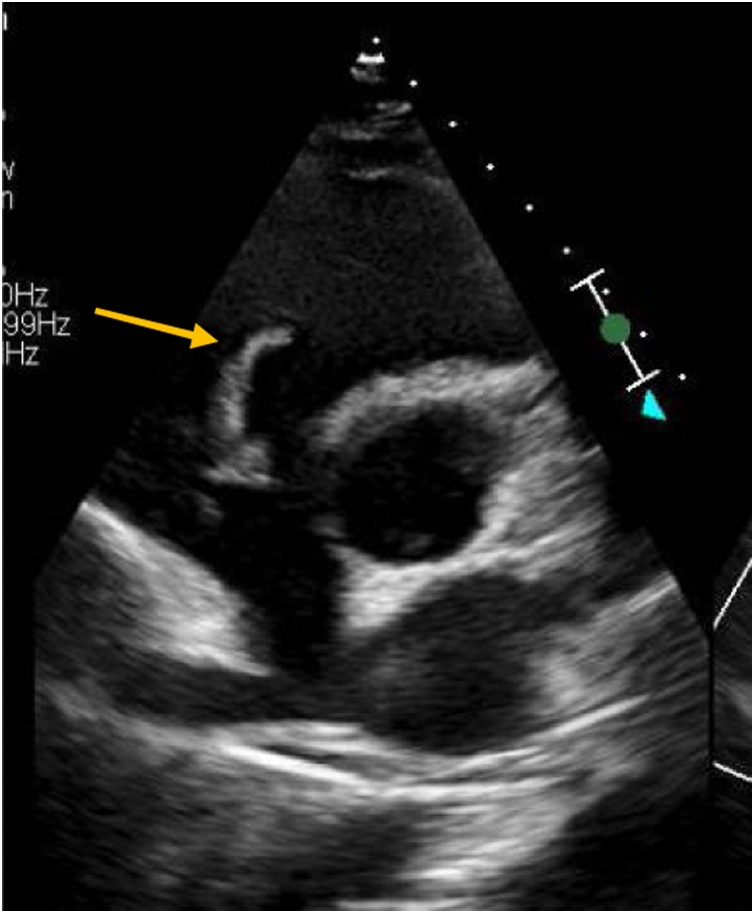
Transthoracic echocardiography parasternal short axis view showing elongated chaotic highly mobile echo density attached to normally structured tricuspid valve.

**Fig. 3:**
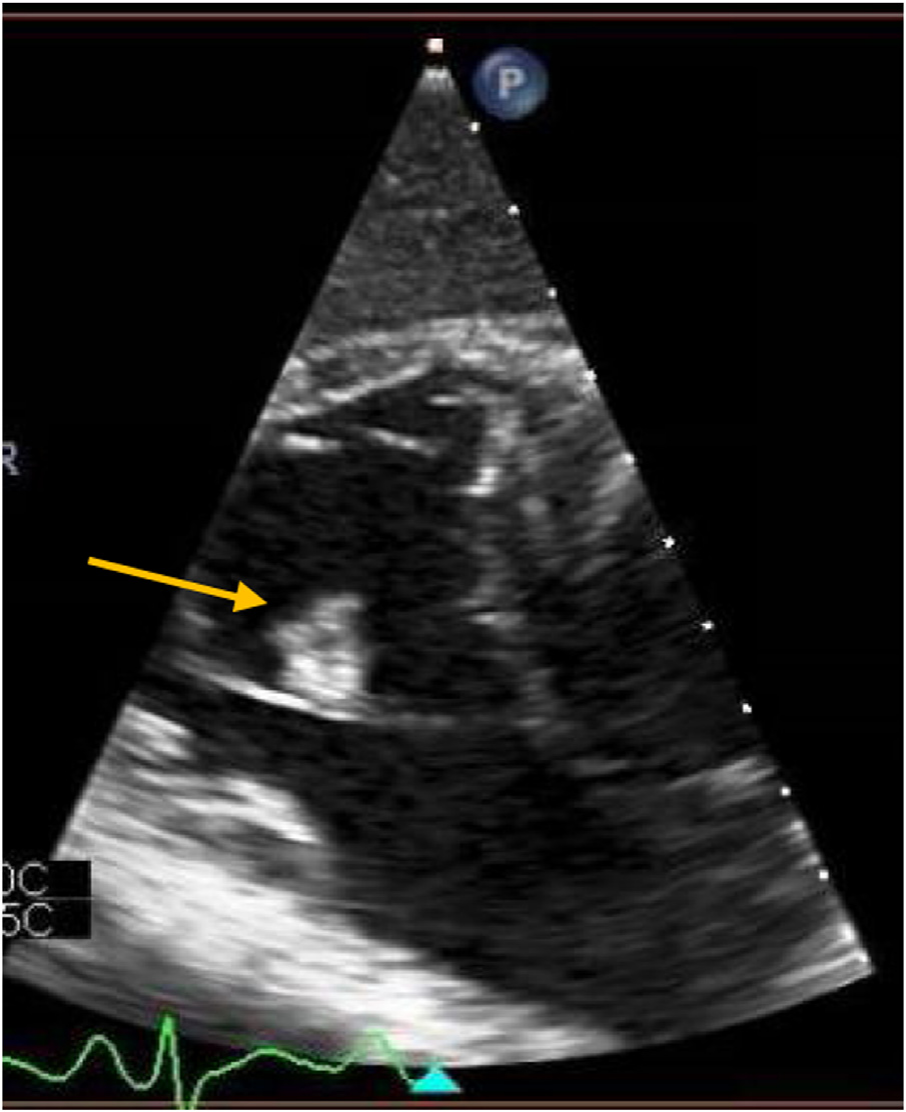
Transesophageal Echocardiography showing rectangular shaped vegetation attached to the entire length of anterior tricuspid leaflet, measuring 1.8 * 1.5 cm.

Transthoracic echocardiography was done to rule out IE given that she has bacteremia with virulent organism, it showed elongated chaotic highly mobile echo density attached to native tricuspid valve with mild tricuspid valve regurgitation. (Fig. 2). Transesophageal echocardiography showed vegetation mass attached to entire length of anterior tricuspid leaflet which is highly mobile with size of 1.5 *1.8 cm (Fig. 3). No defects were noticed in both echocardiograms. She was diagnosed as IE based on modified Dukes criteria, meeting one major criteria; positive echocardiogram, and three minor criteria: fever, positive blood cultures that do not meet major criteria and possible septic pulmonary emboli as CT chest showed bilateral small patchy consolidations.

**Fig. 4:**
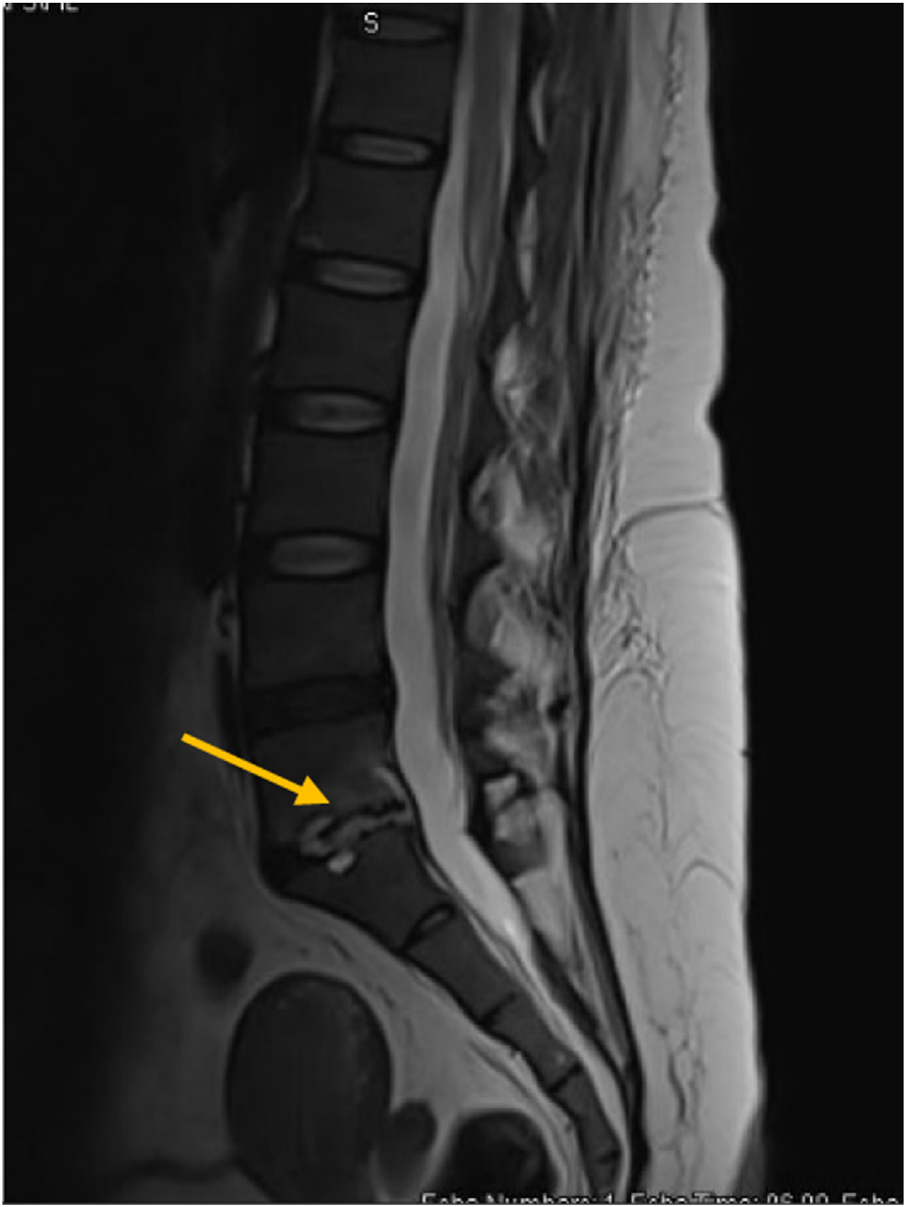
Repeated Lumbosacral MRI showing L5-S1 spondylodiscitis.

Given the recent lumbar epidural injection with the current bacteremia and non-resolving back pian, a local infectious complication was suspected, lumbosacral spine MRI was done and showed L5-S1 spondylodiscitis with inflammatory right posterior paraspinal myositis (Fig. 4).

Patient continued to spike fever and repeated blood cultures showed persistent bacteremia despite being on optimum antimicrobial coverage with teicoplanin and rifampicin for about one week. Eventually, she was referred for urgent surgical management. Cardiothoracic surgery was carried out through conventional median sternotomy incision, right atrium was opened longitudinally then the area was inspected, A large shaggy vegetation was found attached to the anterior leaflet of tricuspid valve, tricuspid leaflets were intact. A well-defined defect was identified between the right atrium and left ventricle at the level of anterior-septal commissure of the tricuspid valve presenting congenital Gerbode defect, its location was confirmed with intraoperative TEE. Vegetation was removed from the anterior leaflet and the Gerbode defect was closed by prolene suture placed underneath adjacent parts of anterior and septal leaflets. Tissue culture of the vegetation showed *S. Lugdunesis growth*.

Post Operation, patient had recovered gradually, and subsequent blood culture showed clearance of bacteremia. she was kept on IV teicoplanin and rifampicin (since the primary source most likely is related to bone and soft tissue infection) for 4 weeks. Her back pain resolved, and she was discharged home on oral Co-trimoxazole and rifampicin for another 2 weeks to complete total 6 weeks of antibiotics.

## Discussion

Staphylococcus lugdunensis is a newly recognized coagulase-negative staphylococcus (CoNS) that was first isolated by Frenet et al. in 1988 [1]. It is considered as a normal skin flora with preferential colonization of the inguinal area [2]. Studies showed that the prevalence of S. lugdunensis colonization may reach up to 30%–50% of patients [3]. CoNS is frequently considered as a contamination, however, S. lugdunensis should be considered as a pathogen, it has similar virulence to S. aureus, both have similar fibrinogen-binding proteins that play an important role in their virulence [4]. The spectrum of diseases caused by S. lugdunensis ranges mainly from skin and soft tissue infections to more serious conditions like bacteremia with subsequent seeding with arthritis, osteomyelitis, and infective endocarditis [5,6].

Approximately 5% of cases of native valve endocarditis are caused by CoNS [7]. S. lugdunensis has been reported as the second most common CoNS, after S. epidermidis, to cause infective endocarditis (IE) [8]. Liu et al. in their literature review of 67 documented cases of S. lugdunensis IE in the period from 1988 to 2008 found that 87 % of cases occurred on the left side valves, and tricuspid valve involvement was reported only in 4 cases (6.0 %). Native valves involvement was reported in 80.6 % of cases [9,10]. Native valve endocarditis caused by CoNS has usually indolent course, however S. lugdunensis results frequently in more aggressive form with multiple complications resembling native valve endocarditis caused by S aureus [11].

The most common complications reported with S lugdunensis IE as found by Anguera et al. in their review of 69 patients are: heart failure (45 %), periannular abscess (19 %), and systemic embolization (30 %) [12]. Furthermore, S. lugdunensis can cause septal destruction resulting in ventricular septal defect (VSD) [13] or in rare cases acquired Gerbode defect [14]. S. lugdunensis IE has high mortality rate reaching up to 38.8 %. Medical treatment alone without surgical intervention was an independent risk factor for mortality [9]. Although most isolates of S. lugdunensis are sensitive to oxacillin due to the lack of mecA gene [15], medical treatment alone is usually not enough and surgical intervention with valve replacement is needed in about 70 % of patients with S. lugdunensis IE [9,15].

Spinal epidural and other paraspinal injections; like Intra-articular facet joint injection, are common procedures for several indications. Infectious complications: such as Spondylodiscitis and paraspinal abscess, are considered uncommon following these low morbid procedures. Diagnosis of spinal epidural abscess is challenging and frequently delayed until solid symptoms develop due to non-specific misinterpreted presentation and absence of clear infection signs at the injection site [16]. Infective endocarditis is even an extremely rare complication of such procedures. In a review of adverse events encountered during 11,980 Intra-articular facet joint steroid injection procedures in the period from 2007 to 2017, only one case of IE was reported [17]. Spondylodiscitis and paraspinal abscesses can be either nidus for bacteremia and IE, as in our case, or as a result of embolic event from IE itself. Detailed history and high index of suspicion is required in case of combined IE and Spondylodiscitis in order to determine which one is the primary source of infection and manage patient properly [16,[18], [19], [20]]).

Gerbode defect is a shunt communicating between the left ventricle and right atrium, it is divided into 2 types; Congenital and acquired. The acquired type can be either iatrogenic; following cardiac surgeries or percutaneous cardiac interventions, or non-iatrogenic; as a complication of infective endocarditis, trauma or myocardial infarction [21,22]. Infective endocarditis accounts for about 28 % of acquired Gerbode defect [23]. On the other hand, preexistent congenital Gerbode defect results in large systolic pressure gradient between the left ventricle and the right atrium which may put the native tricuspid valve under a higher risk of developing infective endocarditis.

In our case, patient developed *S. lugdunesis* bacteremia associated with tricuspid valve IE and L5-S1 spondylodiscitis 5 weeks following a CT guided lumbar epidural injection. We think the likely explanation is that procedure was complicated by local spondylodiscitis which led to bacteremia and hematogenous spread to the tricuspid valve causing infective endocarditis. On the other hand, the tricuspid valve IE could be the primary nidus itself that caused metastatic infection to the spine and resulted in spondylodiscitis. Other possibility could be that patient had another nosocomial infection that is not related to the procedure and which led to bacteremia causing both tricuspid valve IE and spondylodiscitis. However, these possibilities are less likely as the site of the spondylodiscitis and myositis is the same site of the procedure. In addition, systemic emboli are usually caused by left-sided IE rather than right-sided IE which results in septic pulmonary emboli.

## Conclusion

*S. lugdunesis*, a member of CoNS, behaves aggressively and can lead to destructive IE. High index of suspicion even post low risk procedures is essential for early diagnosis and treatment. Surgical therapy is frequently required with *S. lugdunesis* IE and associated with better outcome.

## Author statement

The authors declare that this manuscript is original, has not been published before and is not currently being considered for publication elsewhere.

The authors confirm that the manuscript has been read and approved by all named authors and that there are no other persons who satisfied the criteria for authorship but are not listed. We further confirm that the order of authors listed in the manuscript has been approved by all of us.

The authors understand that the Corresponding Author is the sole contact for the Editorial process. He is responsible for communicating with the other authors about progress, submissions of revisions and final approval of proofs.

## Funding

Open-access publication fees were covered by the Qatar National Library (QNL).

## Consent

Written informed consent was obtained from the patient for publication of this case report and accompanying images. A copy of the written consent is available for review by the Editor-in-Chief of this journal on request.

## Ethical approval

The Medical Research Center of Hamad Medical Corporation has granted permission for this case report to be published, **MRC-04-20-880**.

## Author contribution

• **Osama Nweiran Al-Khalaila:** Study design, Data collection and analysis, writing “Literature Review”.
• **Laith Fawzat Tbishat:** Data collection and analysis, writing “case presentation”.
• **Mohamed Salah Abdelghani:** Writing “Abstract”, “Introduction”, and “Conclusion”.
• **Ahmad A. A. Al Bishawi:** Study design, Data collection and analysis, writing “Literature Review”.
• **Adila Shaukat Kashaf:** Study Design, writing “case presentation”, taking written consent from the patient.
• **Dina Alwaheidi:** Writing “Abstract”, “Introduction”, and “Conclusion”.
• **Abdulwahid Al Mulla:** Study Design, writing “Literature Review”, Final Review.

All authors have approved the final article to be submitted.

## Data availability

Previously reported data were used to support this case report and are available within the manuscript. These prior studies are cited at relevant places within the text as references [[1], [2], [3], [4], [5], [6], [7], [8], [9], [10], [11], [12], [13], [14], [15], [16], [17], [18], [19], [20], [21], [22], [23]].

## Declaration of Competing Interest

The authors report no declarations of interest.
